# 

**Published:** 1842-06

**Authors:** 


### CONTENTS

OF NO. VI.

### ORIGINAL COMMUNICATIONS.

### ESSAYS AND CASES.

ART. I.—Observations on the epidemic Yellow Fever of Natchez, and of the South-west. By JOHN W. MONETTE, M. D., of Washington, Mississippi. - - 401

ART. II.—A Case of Thymic Asthma. By BENJAMIN DENNIS, M. D., of Cincinnati, Ohio. - - 427

### REVIEWS.

ART. III.—The Practice of Medicine; or a Treatise on Special Pathology and Therapeutics. By ROBLEY DUNGLISON, M. D., Professor of the Institutes of Medicine, &c., in Jefferson Medical College, Philadelphia; Lecturer on Clinical Medicine, and attending Physician at the Philadelphia Hospital, &c. &c. In two volumes, pp. 1323. Lea & Blanchard, Philadelphia. - 429

### SELECTIONS FROM AMERICAN AND FOREIGN JOURNALS.

Malformation of the Shoulders. - - - 451

Treatment of Mesenteric Glandular Affections. - 452

Fracture of the Neck of the Femur within the Capsule—Osseous Union. - - - - - 457

Foreign Body in the Air Passage for Nine Months—Expulsion—Cure. - - - - - - 457
Case of Inflamed Hernia. - - - - 458
MALGAIGNE on Pseudo-Strangulation, or Simple Inflammation of Hernia. - - - - - 460
On the Stinging Organs of the Medusæ. - - 463
Recovery from Tubular Pregnancy by the Artificial Production of Abortion. - - - - - 464
Tobacco in Hysteria. - - - - - 465
Idiopathic Hydrophobia. - - - - 467

ORIGINAL INTELLIGENCE.

Medical Convention of Ohio. - - - - 469
Western Periodicals. - - - - - 472
Medical Schools of the West. - - - - 473
Death of Dr. Blythe. - - - - - 474
Erratum. - - - - - - - 476

